# Supplementary material for: Regularized Joint Reconstruction and Slab Combination for Accelerated Three‐Dimensional Multi‐Slab Diffusion‐Weighted Imaging Using Multi‐Scale Energy Models
Source: Magn Reson Med. 2026 Jul 28;96(5):2321–35. doi: 10.1002/mrm.70512 (PMC13527291; doi:10.1002/mrm.70512)
Supplement: Supplementary file 1 [file MRM-96-2321-s001.pdf]

S1 ENERGY MODEL TRAINING DETAILS

The energy model in EPEN is trained via denoising score matching (DSM) [1, 2] over a bounded noise range sufficient for regularization, rather than the full noise schedule required for generative diffusion models. A diffusion model must learn the score from near-zero noise, up to pure Gaussian noise, because generation proceeds by progressively denoising pure noise through every scale, requiring tens to hundreds of sequential network passes at inference. Because EPEN uses the prior solely as a MAP regularizer and never for generative sampling, the bounded training range and small number of score evaluations per reconstruction mean that EPEN retains the computational and training-data footprint of a standard CNN-based regularized reconstruction rather than that of a diffusion model.

Training references were obtained by spacing slabs so that each slab's extended-FOV margin met that of the neighboring slab, suppressing slab cross-talk at acquisition. Central slices were extracted from each slab where the RF excitation profile is approximately uniform, and the boundary slices were discarded. This combination of physical inter-slab gap and boundary-slice removal yields well-separated, artifact-free slices. Since noise-free measurements are unavailable, references were denoised using Marchenko-Pastur Principal Component Analysis (MPPCA) [3], chosen for its established role in dMRI preprocessing and its parameter-free, noise-distribution-agnostic operation.

For score-based models, training on denoised rather than noisy references is important since the DSM objective would otherwise learn the score of the noisy distribution rather than the clean-image prior, a limitation documented in recent score-model literature [4]. Because MPPCA is a fixed classical denoiser, the achievable reconstruction quality is bounded by the quality of its output; learned or self-supervised alternatives such as SURE-Score [4], and Noise2Noise [5] are not constrained by MPPCA's assumptions and could, in principle, yield higher-quality references, at the cost of paired acquisitions or explicit noise modeling. Optimizing this denoising step is a direction for future work.

S2 NETWORK ARCHITECTURE AND COMPUTATIONAL DETAILS

The energy network is a DRUNet comprising four downsampling and four upsampling stages with 64, 128, 256, and 512 feature channels, respectively, each incorporating four residual blocks and connected via skip connections. The network contains 32 million parameters, which is compact relative to the 50–114 million parameters typically used in denoising diffusion models with comparable architectures [6]. Complex-valued inputs are handled as two separate channels [7].

In terms of computational cost, EPEN reconstructed a single diffusion-weighted volume in approximately 5.3 minutes using 3 outer alternating iterations with 20 iterations per image-update sub-problem, on an NVIDIA A100 40 GB GPU. For comparison, NPEN used 10 outer Gauss–Newton iterations with 50 inner CG iterations, requiring approximately 4.2 minutes per volume on the same hardware. All timings were measured using a representative in-vivo dataset (Dataset 1, 14 k_z).

S3 SYNTHETIC DATASET GENERATION

To simulate the 3D multi-slab acquisition, the slab excitation profile was extracted from an MRI phantom experiment, where a slab thickness of 14 mm and a slab FOV of 20 mm (40% oversampling) was prescribed, and 20 k_z encodes were employed to sample the FOV fully. The excitation and refocusing pulses were designed using the Shinnar-LeRoux algorithm [8] with a time-bandwidth product of 12; the durations of the excitation and refocusing pulses were 5.1 and 17.9 ms, respectively. The slab profile of the central slab from the phantom experiment was estimated by dividing the single-slab image by the sum-of-squares and was treated as a representative template tiled axially across all slab positions to generate slabs with 20% extended-FOV margin. Three cases were studied: a fully sampled case (20 k_z) and two accelerated cases (14 k_z and 10 k_z).

Table S1. Denoising performance of the EPEN energy model trained with different numbers of training slices, evaluated on a held-out validation set. Metrics are computed against the MPPCA-denoised references. All trained models substantially improve over the noisy input, and performance degrades only gradually as the training set is reduced.

Training samples	PSNR	SSIM
Noisy input (baseline)	20.84 ± 0.25	0.2049 ± 0.06
∼1600 (30%)	31.65 ± 1.26	0.6570 ± 0.04
∼2600 (50%)	31.73 ± 1.34	0.6714 ± 0.04
∼3600 (70%, used in paper)	32.22 ± 1.61	0.7208 ± 0.03

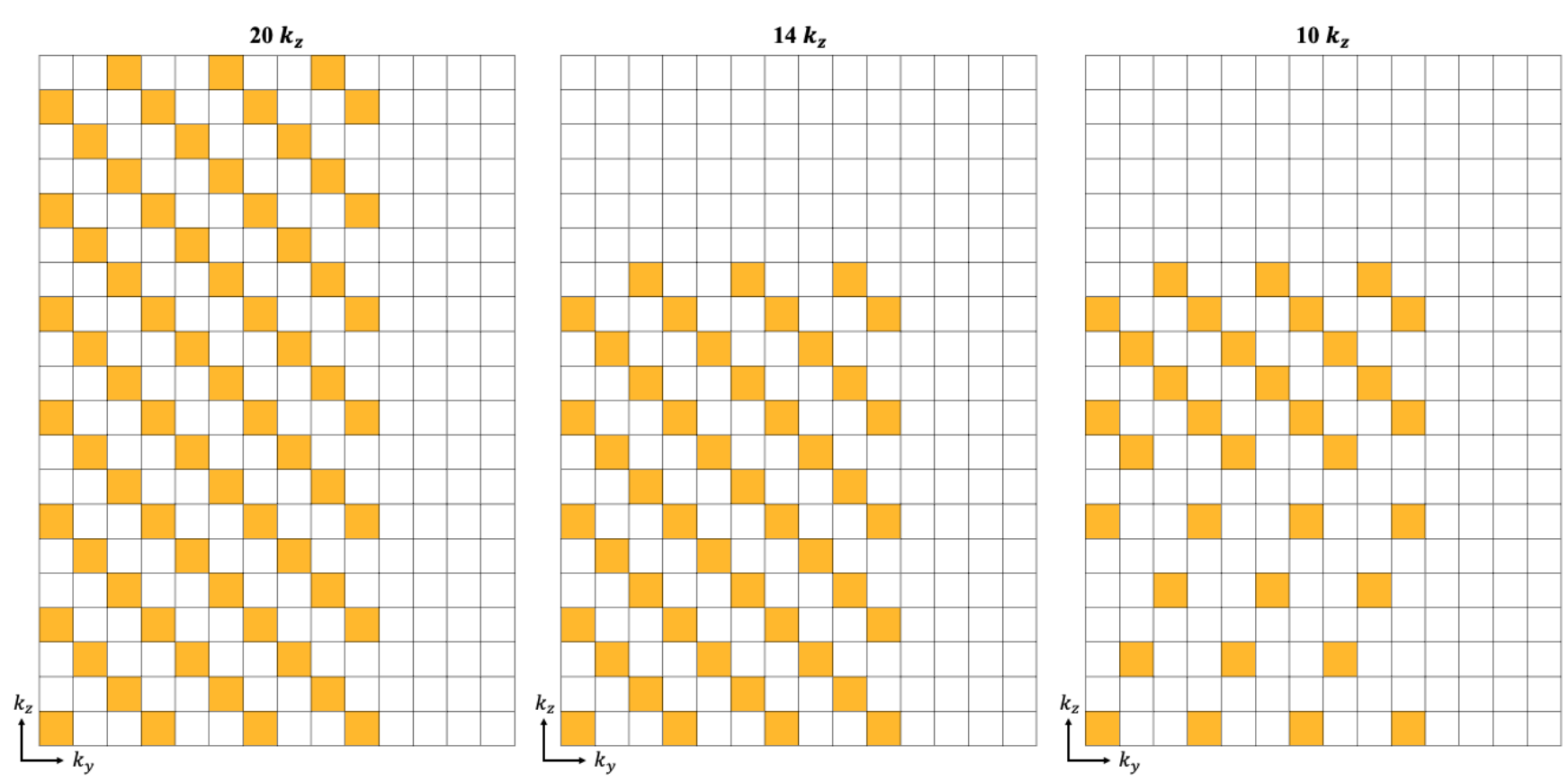

Figure S1. The sampling pattern used for the fully sampled 3D slab acquisition is shown in (a) for the 20 k_z case. Here, a 2D CAIPI sampling pattern is achieved using a shot-selective method with 3 EPI shots. For the accelerated cases, retrospective under-sampling was performed by removing selected k_z encodes from the fully sampled case. (b) shows the 14 k_z case and (c) shows the 10 k_z case.

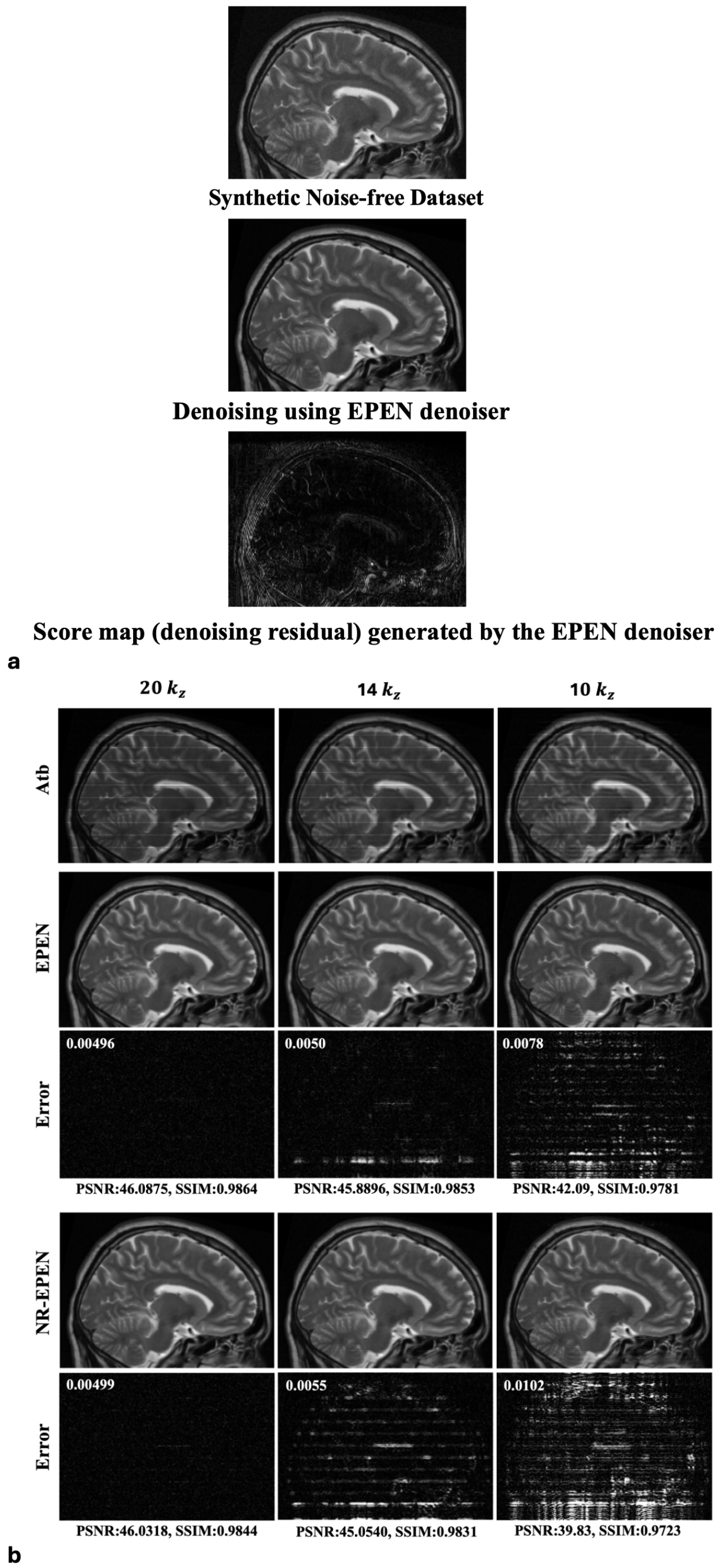

Figure S2. (a) The synthetic noise-free dataset generated using a high-resolution T2-weighted image from the MNI-Colin27 multimodal brain atlas and example denoising results from the EPEN model. To simulate the 3D multi-slab acquisition, the same procedure to generate the DWI synthetic dataset was applied to generate 12 slabs with 20% extended-FOV margin. The energy model, trained only on diffusion data, generalizes well to T2-weighted images. The bottom row shows the learned score (denoising residual), which represents the gradient field the model uses to guide the reconstruction toward the learned data distribution. (b) The proposed regularized slab-combination is demonstrated on simulated noise-free data at (i) 40% oversampling ($20k_z$), (ii) no oversampling (14 k_z), and (iii) 30% under-sampling (10 k_z), and its comparison to an ablated method with no regularization. This controlled setting, which is selected to be noise-free, single-channel, known slab profiles, isolates the contribution of the learned energy prior from confounds such as measurement noise, profile-estimation error, and coil-sensitivity diversity. The error maps are amplified by a factor of 20 to reveal residual artifacts, and the RMSE values are shown in the left-hand corner. The regularized reconstruction is shown to provide robust reconstructions compared to the non-regularized version, with known slab profiles.

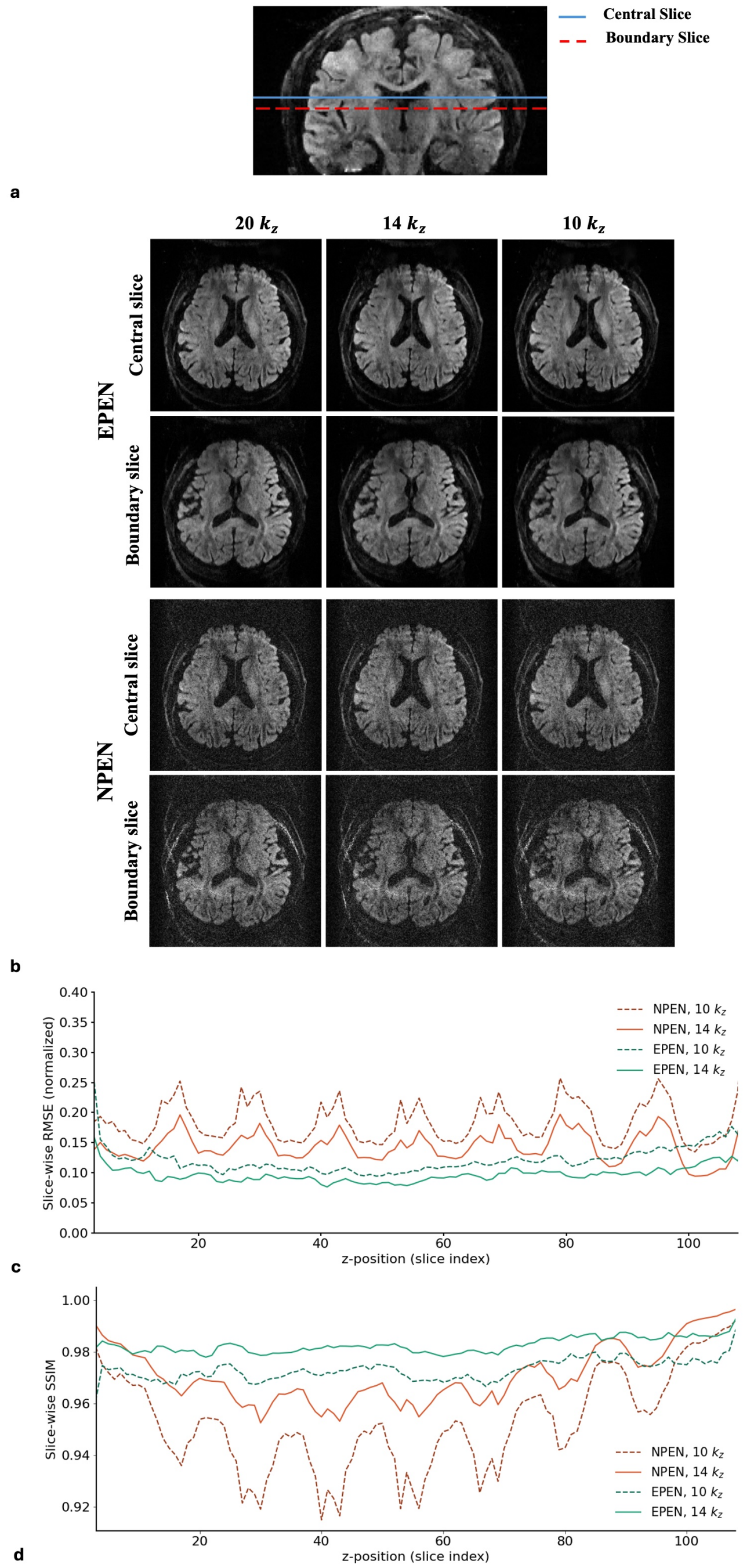

Figure S3. Comparison of image-quality degradation between selected slab-central and slab-boundary slices under k_z undersampling. (a) Coronal localizer with horizontal markers indicating the central (solid) and boundary (dashed) slice positions used for the comparison. (b) Axial views of representative central and boundary slices reconstructed using EPEN and NPEN at 20 k_z, 14 k_z, and 10 k_z. NPEN shows pronounced intensity inhomogeneity and residual structure concentrated at boundary positions that worsen with acceleration, while central slices are comparatively preserved, whereas EPEN maintains consistent image quality across both central and boundary positions. (c) Quantitative slice-wise RMSE plotted against z-position, computed relative to each method's own 20 k_z reference. NPEN exhibits clear peaks at every slab interface that grow with acceleration, while EPEN remains nearly flat, confirming that the joint regularized formulation neutralizes the boundary-specific vulnerability. (d) Quantitative Slice-wise SSIM plotted against z-position, computed relative to each method's own 20 k_z reference. NPEN curves exhibit pronounced periodic SSIM drops at every slab boundary that deepen with acceleration and additionally show reduced SSIM in central slab regions, whereas EPEN curves remain near unity across the entire z-axis at both 14 k_z and 10 k_z, with only minimal residual dips at slab interfaces.

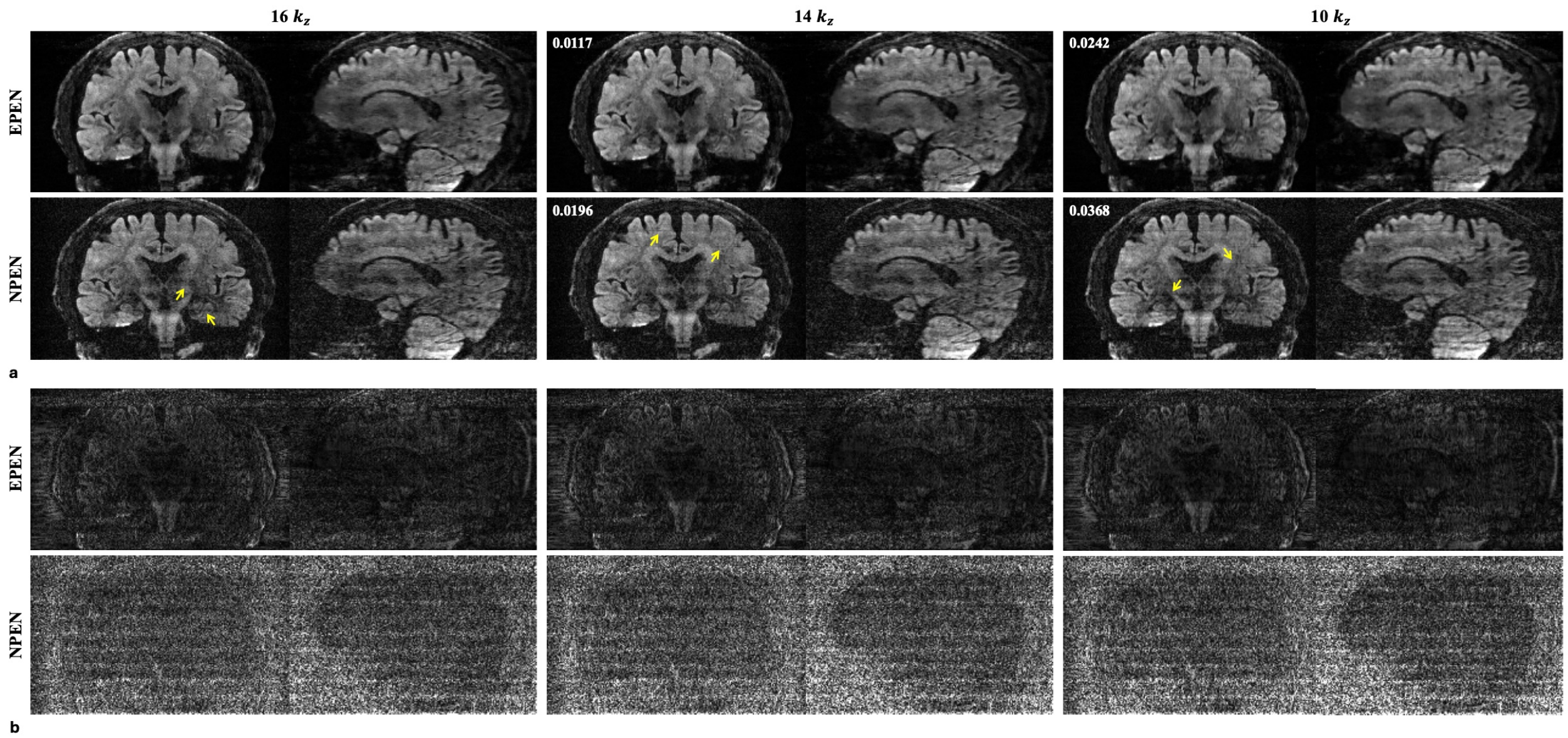

Figure S4. Reconstruction comparison using dataset acquired with 16 k_z. (a) Sagittal and coronal views of reconstructions from EPEN and NPEN at the two undersampled k_z. The RMSE values are shown in the upper left-hand corner of the reconstructed images, and the yellow arrows indicate residual slab-boundary artifacts, showing that these artifacts become more pronounced with increasing undersampling in NPEN, whereas EPEN continues to effectively suppress them even at higher undersampling rates; (b) Corresponding score maps. The 16 k_z acquisition ($\sim$ 14% oversampling) provides a more practical reference baseline than the 20 k_z reference used in the main experiments. Across the comparisons, EPEN outperforms NPEN at both 14 and 10 undersampled k_z, as observed in the main 20 k_z experiments, which confirms that the EPEN advantage does not depend on the choice of reference baseline.

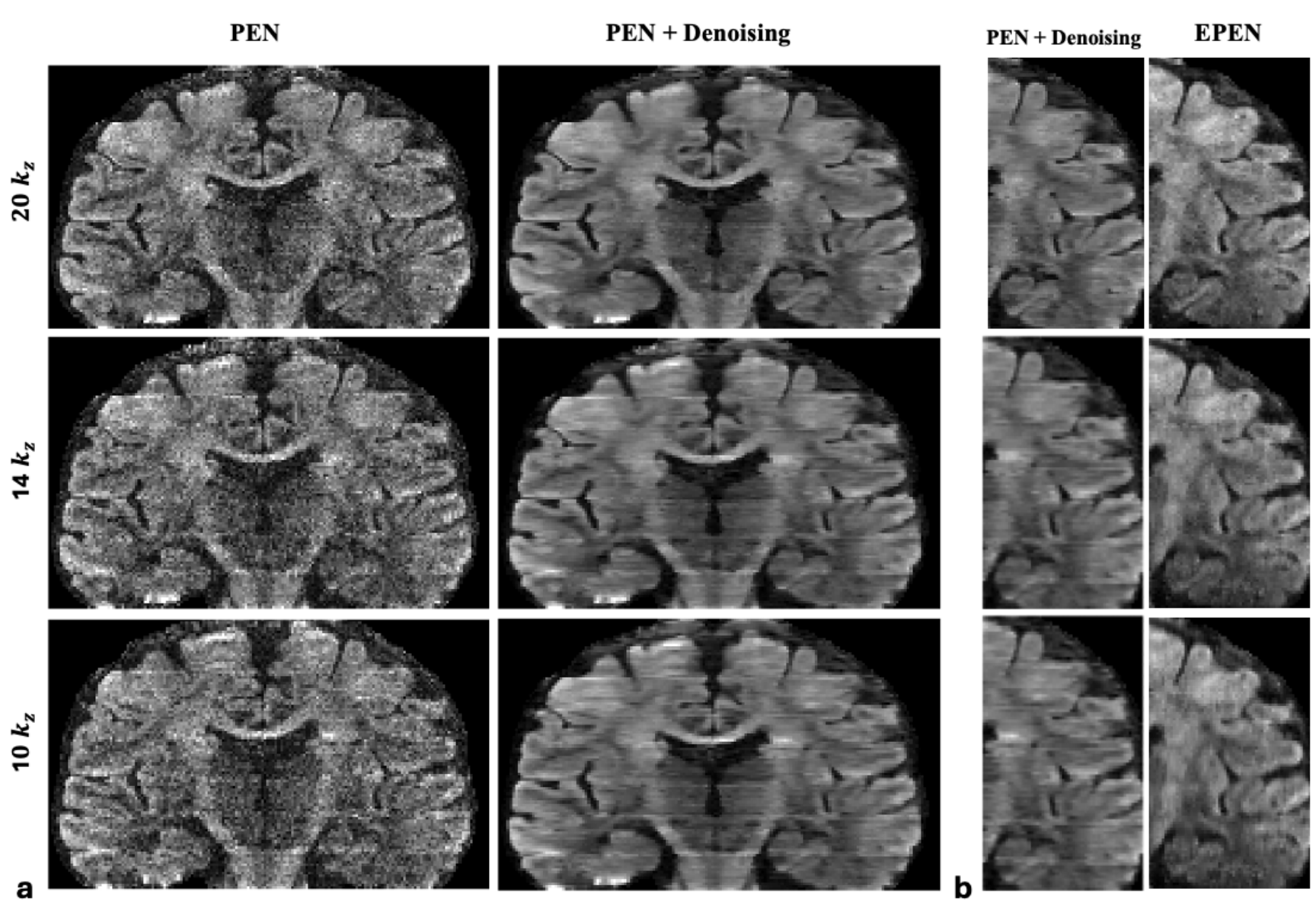

Figure S5. Post-denoising of PEN reconstruction using the learned CNN-based denoiser. Although the images are denoised, the artifacts cannot be removed in this post denoising (a). A selected region of the denoised PEN reconstruction and its corresponding region from the EPEN reconstruction (b).

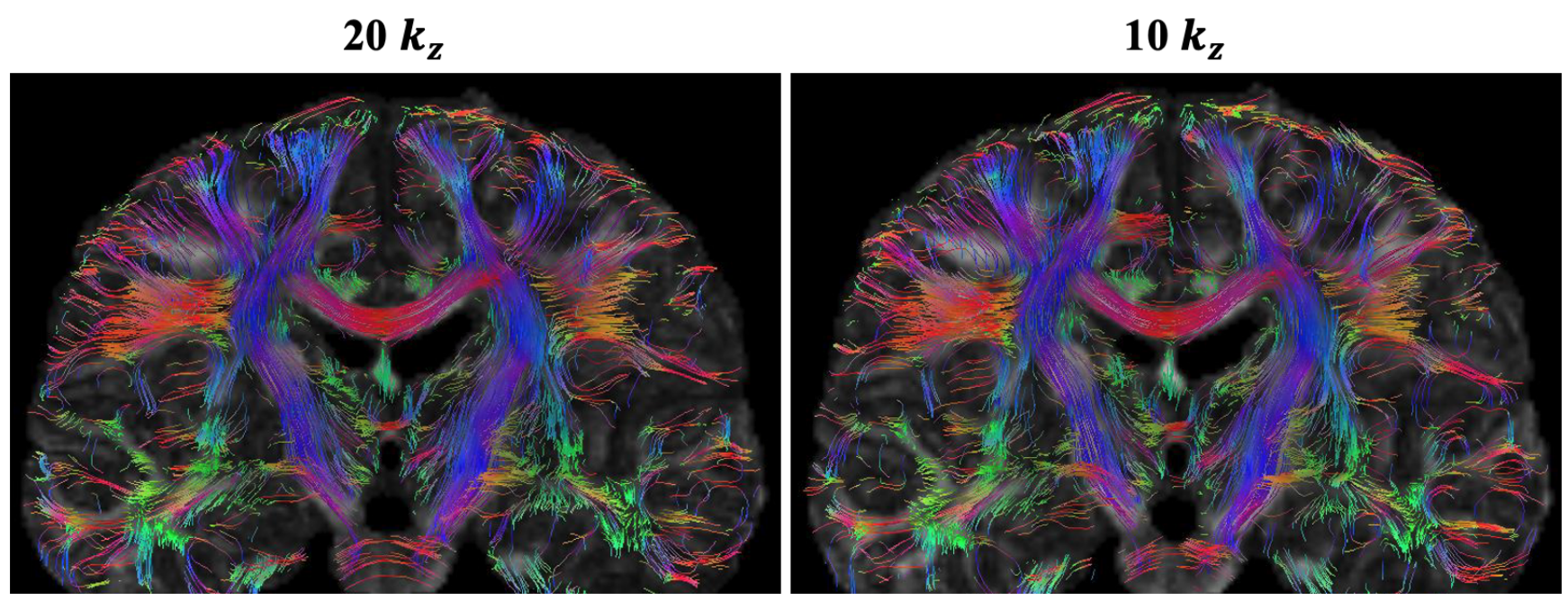

Figure S6. Comparison of fiber tractography from EPEN reconstructions at N_{kz}=20 and 10, using same tracking parameters. The slice direction does not show visible deterioration in the quality of the fiber tracts.

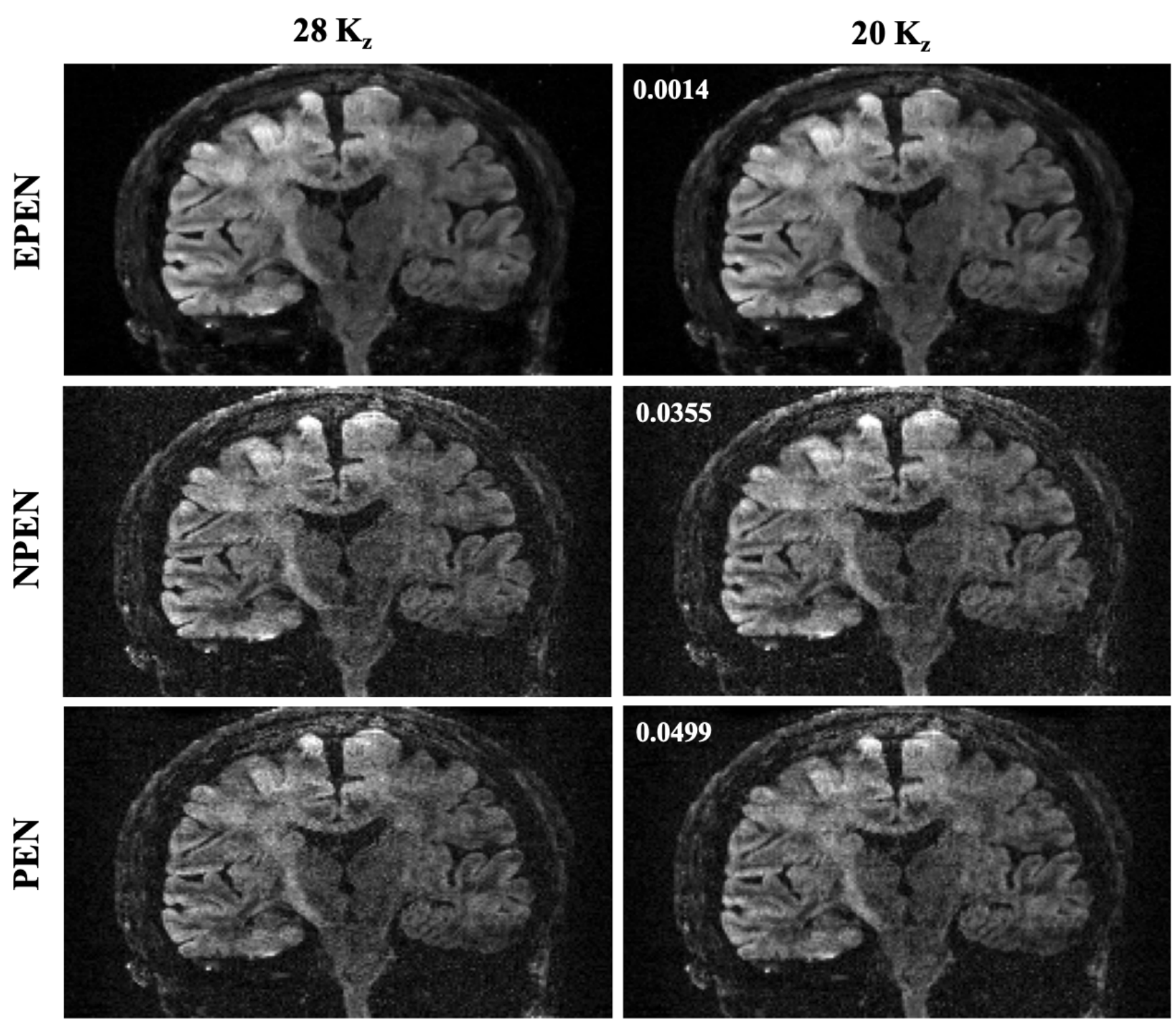

Figure S7. The reconstruction results for the in-vivo dataset 5 with doubled phase encode FOV 28k_z, and 20k_z undersampling.

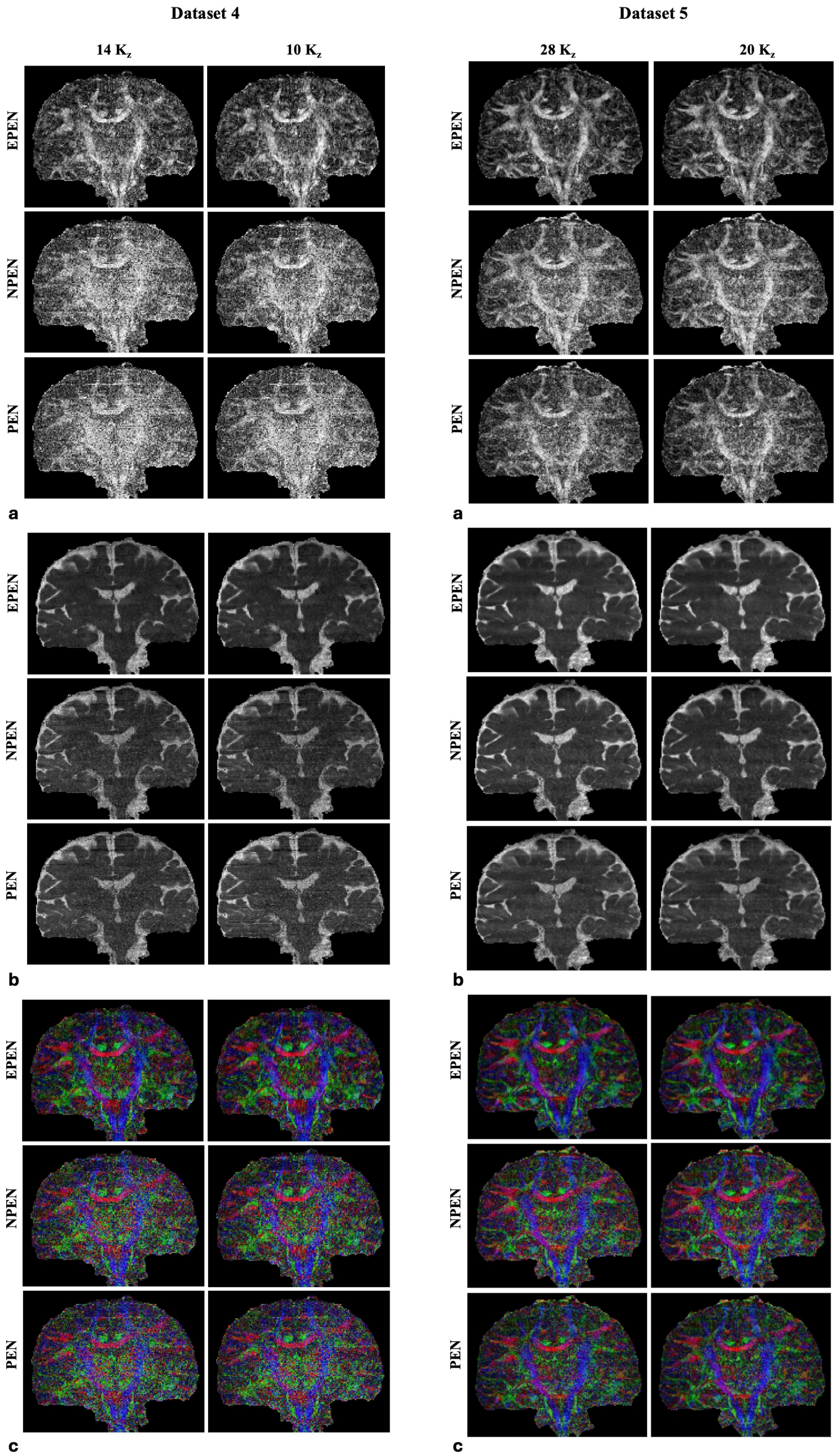

Figure S8. DTI analysis of the reconstruction results for the in-vivo dataset 4 (left) and 5 (right).

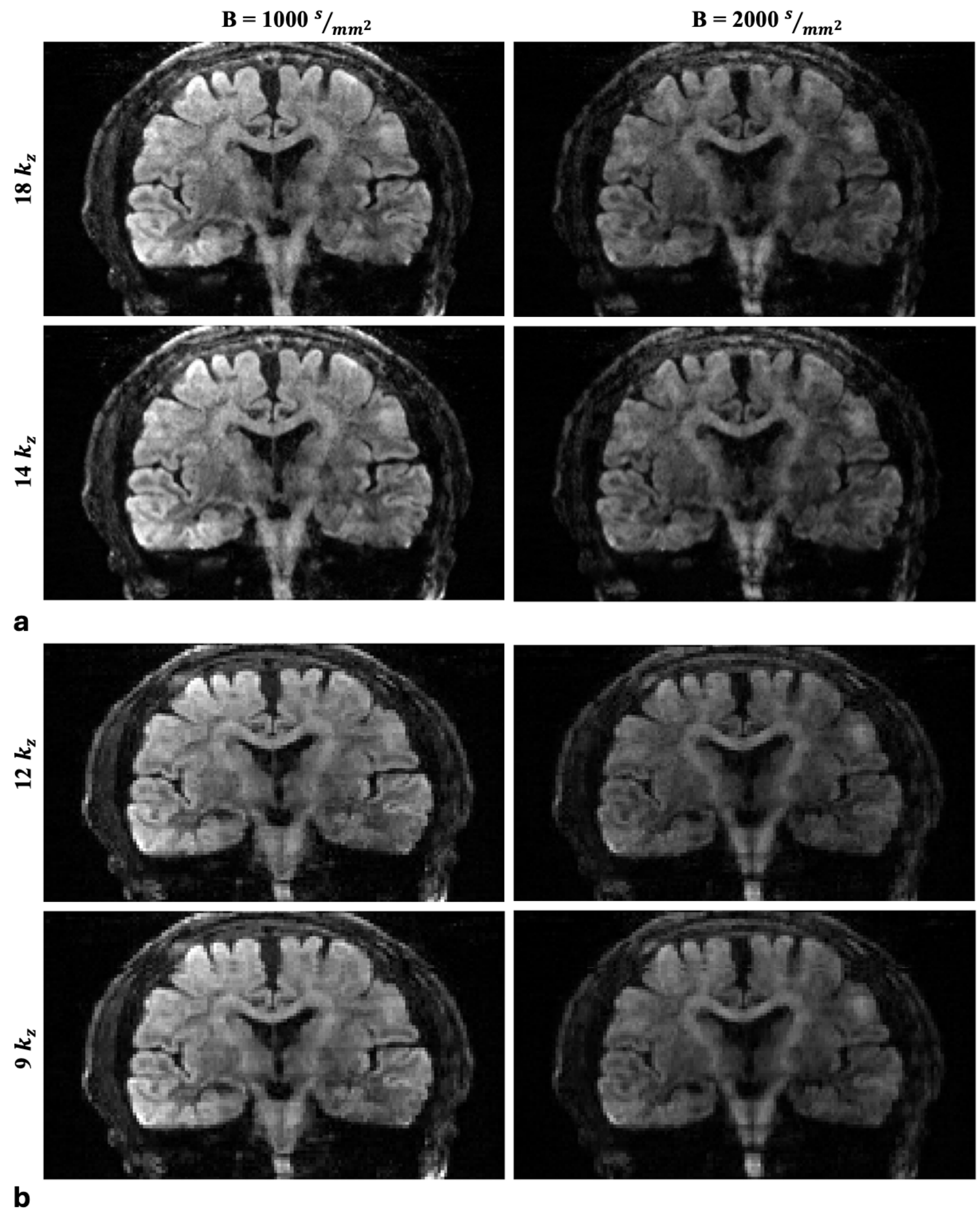

Figure S9. EPEN generalizability across spatial resolutions and diffusion weightings. Reconstructions are shown for datasets 6 and 7 acquired at (a) 1 mm and (b) 1.5 mm isotropic resolution, with b = 1000 s/mm^2 and b = 2000 s/mm^2. All four reconstructions were obtained using the same energy-model checkpoint trained on the original training described in Section 2.3.1; no retraining or fine-tuning was performed for these new acquisitions. EPEN produces stable, slab-boundary-corrected reconstructions across all four configurations, demonstrating that the learned prior generalizes across substantial changes in spatial resolution and diffusion weighting without requiring additional training data.

REFERENCES

1. Zengyi Li, Yubei Chen, and Friedrich T Sommer. Learning energy-based models in high-dimensional spaces with multiscale denoising-score matching. *Entropy*, 25(10):1367, 2023.
2. Pascal Vincent. A connection between score matching and denoising autoencoders. *Neural computation*, 23(7): 1661–1674, 2011.
3. Jelle Veraart, Dmitry S Novikov, Daan Christiaens, Benjamin Ades-Aron, Jan Sijbers, and Els Fieremans. Denoising of diffusion mri using random matrix theory. *Neuroimage*, 142:394–406, 2016.
4. Asad Aali, Marius Arvinte, Sidharth Kumar, and Jonathan I Tamir. Solving inverse problems with score-based generative priors learned from noisy data. In *2023 57th Asilomar Conference on Signals, Systems, and Computers*, pages 837–843. IEEE, 2023.
5. Jaakko Lehtinen, Jacob Munkberg, Jon Hasselgren, Samuli Laine, Tero Karras, Miika Aittala, and Timo Aila. Noise2noise: Learning image restoration without clean data. *arXiv preprint arXiv:1803.04189*, 2018.
6. Jonathan Ho, Ajay Jain, and Pieter Abbeel. Denoising diffusion probabilistic models. *Advances in neural information processing systems*, 33:6840–6851, 2020.
7. Jyothi Rikhab Chand and Mathews Jacob. Multi-scale energy (muse) framework for inverse problems in imaging. *IEEE Transactions on Computational Imaging*, 10:1250–1265, 2024. doi: 10.1109/TCI.2024.3449101.
8. John Pauly, Patrick Le Roux, Dwight Nishimura, and Albert Macovski. Parameter relations for the shinnar-le roux selective excitation pulse design algorithm (nmr imaging). *IEEE transactions on medical imaging*, 10(1):53–65, 1991.
